# Induction of the vascular endothelial growth factor pathway in the brain of adults with fatal falciparum malaria is a non-specific response to severe disease

**DOI:** 10.1111/j.1365-2559.2010.03619.x

**Published:** 2010-08

**Authors:** Isabelle M Medana, Nicholas P J Day, Rachel Roberts, Navakanit Sachanonta, Helen Turley, Emsri Pongponratn, Tran Tinh Hien, Nicholas J White, Gareth D H Turner

**Affiliations:** 1 Nuffield Department of Clinical Laboratory Sciences, John Radcliffe HospitalOxford, UK; 2 Centre for Clinical Vaccinology and Tropical Medicine, Churchill HospitalOxford, UK; 3 Mahidol Oxford Research Unit, Mahidol UniversityBangkok, Thailand; 4 Department of Tropical Pathology, Faculty of Tropical Medicine, Mahidol UniversityBangkok, Thailand; 5 Centre for Tropical Diseases, Cho Quan HospitalHo Chi Minh City, Viet Nam

**Keywords:** cerebral, hypoxia, immunohistochemistry, malaria, pathophysiology, *Plasmodium falciparum*

## Abstract

Medana I M, Day N P J, Roberts R, Sachanonta N, Turley H, Pongponratn E, Hien T T, White N J. & Turner G D H (2010) *Histopathology***57**, 282–294

**Induction of the vascular endothelial growth factor pathway in the brain of adults with fatal falciparum malaria is a non-specific response to severe disease**

**Aims:**

Pathological or neuroprotective mechanisms in the brain in severe malaria may arise from microvascular obstruction with malaria-parasitized erythrocytes. This study aimed to investigate the role of hypoxia and induction of the vascular endothelial growth factor (VEGF) pathway in the neuropathophysiology of severe malaria.

**Methods and results:**

Immunohistochemistry was performed on post mortem brain tissue sections from 20 cases of severe malaria and examined for the expression of transcriptional regulators of VEGF [hypoxia-inducible factor-1 alpha (HIF-1α), HIF-2α], DEC-1, VEGF, VEGF receptors 1 and 2, and the activated, phosphorylated VEGF receptor 2 (pKDR). HIFs showed limited protein expression and/or translocation to cell nuclei in severe malaria, but DEC-1, which is more stable and regulated by HIF-1α, was observed. There was heterogeneous expression of VEGF and its receptors in severe malaria and non-malarial disease controls. pKDR expression on vessels was greater in malaria cases than in controls but did not correlate with parasite sequestration. VEGF uptake by malaria parasites was observed.

**Conclusions:**

VEGF and its receptor expression levels in severe malaria reflect a non-specific response to severe systemic disease. Potential manipulation of events at the vasculature by the parasite requires further investigation.

## Introduction

Falciparum malaria, a mosquito-borne disease caused by the protozoan parasite *Plasmodium falciparum*, remains a major global health problem, causing an estimated 1–2 million deaths each year worldwide. A common and frequently lethal manifestation of severe malarial disease is cerebral malaria (CM), characterized by decreased consciousness, coma and fitting, which resembles a metabolic or anaesthetic encephalopathy.^1^ The overall mortality of adult CM is about 15–20% but depends on associated vital organ dysfunction. In patients with ‘pure’ CM and no other complications of severe malaria the mortality is 8%, which rises towards 50% with associated acute renal failure and acidosis. Most deaths occur within 48 h of admission. Full recovery of consciousness takes a median of 2 days in patients with a Glasgow coma score of <11, but occasional adult patients may take >1 week to recover consciousness.^2^ It has been suggested that coma may be neuroprotective. Neurons stressed by an inadequate supply of oxygen and nutrients, and an unfavourable metabolic milieu, may protect themselves by reducing energy demands. Premature reversal of coma might therefore increase the risk of neuronal damage.^3^

The histopathological hallmark of CM is engorgement of cerebral capillaries and venules with parasitized red blood cells (PRBC) and non-parasitized RBCs. Our studies of the pathology of CM in southeast Asian adults indicate that sequestration of *P. falciparum*-infected red blood cells in the microvessels of the brain, and resulting microvascular obstruction, is a key initiating factor in pre-mortem coma.^4^–^6^ Understanding how sequestration initiates coma, and subsequent neuropathological processes occurring in the brain, presents an important question in the search for adjuvant therapy in severe disease. It is also likely that a number of systemic factors, such as immune responses or metabolic changes due to systemic effects of disease, may modulate or contribute to coma and neuropathological injury (reviewed in^7^). However, from our comparative studies in human immunodeficiency virus encephalitis and progressive multifocal leukoencephalopathy,^8^ it is clear that the amount of tissue destruction and inflammation is considerably less in adult southeast Asian CM patients. A potential survival strategy of the parasite would be to limit the amount of vascular damage that would attract inflammatory cells and subsequently harm developing gametocytes present within the sequestered vessel.

The relationship between parasite sequestration, signalling events across the blood–brain barrier and subsequent neuropathological or protective responses within the brain parenchyma in severe malaria is poorly understood. Considering the prominent vascular obstruction it is unclear why there is not more irreversible injury and inflammation. One hypothesis is that parasite sequestration in cerebral microvessels leads to local hypoxia and increased expression of hypoxia-inducible gene products such as vascular endothelial growth factor (VEGF) and its receptors. The VEGF signalling pathway is known to activate a range of signals that are cytoprotective, angiogenic, and neurotrophic.^9^ Two studies examining serum and cerebrospinal fluid (CSF) levels of VEGF have recently been reported in African children with malaria. One study of a small group of Ghanaian children (*n* = 19) showed no difference between CM, severe malarial anaemia and non-malarial disease at the time of death.^10^ In a study of Kenyan children (*n* = 124), serum and CSF levels of VEGF were not associated with the development of neurological sequelae but, rather, features associated with a poor outcome such as seizures, raised intracranial pressure and papilloedema.^11^ Serum VEGF levels were investigated in adult and paediatric patients from India, of whom 25 were healthy controls, 48 had mild malaria and 60 had CM (48 survived and 12 died).^12^ CM patients who died had a significantly lower level of VEGF compared with the other patient groups, which led to the authors to suggest that VEGF should be considered for adjunctive therapy to improve the treatment outcome in CM. In a more recent study of 146 adults from Papua Indonesia there were no associations between elevated VEGF concentrations and disease severity or a poor outcome.^13^ In this study plasma VEGF levels were lower in severe malaria compared with patients with moderately severe malaria or healthy controls and VEGF was inversely associated with lactate.^13^

Only two studies have investigated VEGF protein expression in the brain of severe or CM cases.^14^,^15^ In the first study, immunohistochemical analysis of VEGF was carried out in the brain of six European adults who had died from CM.^14^ VEGF was found in astrocytes and VEGF receptor 1 (VEGFR1; flt-1) in Dürck’s granulomas. Localization of KDR and the extent of receptor activation and phosphorylation were not determined. The more recent study by our group^15^ showed the unexpected finding of VEGF immunolabelling by sequestered malaria parasites. Although parasite immunolabelling was not reported in the first study, we believe the findings to be of significance. VEGF uptake was confirmed *in vitro*^15^ and in a clinical study by Yeo *et al*.^13^ an inverse association was observed between plasma VEGF concentrations and parasite biomass, suggesting potential manipulation of the host response to malaria infection by the parasite.

The aims of this study were to define the endogenous expression of VEGF and its (activated) receptors in the brain of cases of fatal severe malaria and to identify potential systemic and local regulatory factors. Comparisons were made with a group of control cases characterized by non-infectious, multi-organ failure to ensure differentiation of features specific to malaria compared with a background of agonal neuropathology. Hypoxia and the transcriptional complex hypoxia-inducible factor (HIF) are key regulators of VEGF, so we have investigated the protein expression of HIF-1α and HIF-2α. However, due to the short half-lives of HIFs (a few minutes under normoxia) we have also investigated the expression of another transcriptional protein, DEC-1, which is also induced by hypoxia and HIF-1α but has an estimated half-life of 30 h. In light of the findings of VEGF uptake by parasites we have investigated whether this could affect protein expression levels or the abundance of receptors at the vasculature or within the brain parenchyma. Treating patients with exogenous VEGF may exacerbate inflammation and provide trophic support for the parasite that could potentially worsen the treatment outcome in adults with CM.

## Materials and methods

### Case selection

Autopsy brain specimens were taken from adult patients who had died of severe falciparum malaria on the Malaria Ward, Centre for Tropical Diseases (Ho Chi Minh City, Vietnam), as described previously.^16^ These patients were divided into two groups: CM (*n* = 7) and non-CM (*n* = 13). CM was defined according to World Health Organization (WHO) guidelines on the basis of a Glasgow coma score of ≤11,^3^ other causes of unconsciousness having been excluded (e.g. hypoglycaemia, meningitis or other encephalopathy), by clinical, biochemical and CSF examination. Non-CM patients were those dying from severe malaria without coma, who had a range of clinical features typical of other vital organ system complications.

Control cases were from different causes of death in patients undergoing autopsies at the John Radcliffe Hospital (Oxford, UK). Control sections used to assess the protein expression of VEGF and its receptors in brain came from fatal cases with multi-organ dysfunction that did not have obvious structural neuropathology on standard histological examination (see Table 1). Positive controls for HIF immunostaining included sections of clear cell carcinoma and global hypoxic-ischaemic injury. Positive controls for VEGF and its receptors included sections of normal ovary. Protocols for tissue sampling, storage and use were approved by the Ethical and Scientific Committee of the Centre for Tropical Diseases, Vietnam, OXTREC 029-02, COREC (C01.002) and the Human Tissue Authority license number 12217.

**Table 1:** Control patient data

Patient	Clinical history
MODC1	Pneumonia, pulmonary embolus, deranged blood clotting (International normalized ratio >15), peripheral vasculopathy (critical ischaemia of both feet), renal failure, cardiac arrest due to left ventricular myocardial infarction
MODC2	Rheumatoid arthritis, non-insulin-dependent diabetes mellitus, gastric ulcer, anaemia, heart failure, bilateral bronchopneumonia, preterminal shock with pulmonary oedema and acute tubular necrosis
MODC3	Congestive cardiac failure, acute myocardial infarction, severe generalized atherosclerosis, deteriorating renal function prior to death
MODC4	Empyema, bronchopneumonia and pleural fibrosis
MODC5	Renal transplant, circulatory collapse due to acute ruptures, aortic dissection with pleural and pericardial haematoma

### Immunohistochemistry

Three areas of the brain were examined to judge the patterns of histological immunoreactivity. These included sections from cortex (temporal), diencephalon (or interbrain) situated between the cerebrum and brainstem including the thalamus and projections of the internal capsule, and brainstem (medulla). Immuno-histochemistry was performed with multiple antibodies, with varying methods of antigen retrieval, as detailed in Table 2. The conditions and dilutions of antibodies were optimized in previous experiments or according to manufacturers’ instructions. All sections were from formalin-fixed paraffin-embedded blocks, which were deparaffinized and re-hydrated using graded alcohols to water, and immunostained according to standard protocols. Negative controls included omission of primary and secondary antibodies.

**Table 2:** Details of the antibodies, dilutions, and antigen retrieval methods used in this study

Primary antibody	Specificity	Antigen retrieval	Dilution/incubation time	Source	Reference
ESEE 122	HIF-1α	0.1 m EDTA 3 min, PC	Neat 90 min, RT	In house	27
EP 190b	HIF-2α	0.1 m EDTA 60°C, ON	Neat 90 min, RT	In house	27
CW27	DEC-1	Citrate buffer 10 min, MW	1:1000 45 min, RT	Prof. Adrian Harris, CRUK	18
VG-1	VEGF	0.05 m Tris/0.2 mm EDTA 3 min, PC	1:4, 90 min, RT	In house	28
C-17	VEGF-R1	As above	1:200 90 min, RT	Santa Cruz, CA	
Y-23	VEGF-R2	As above	1:200 90 min, RT	Santa Cruz, CA	
pKDR34	pKDR	Citrate buffer 3 min, MW	1:5 90 min, RT	In house	29

All primary antibodies were visualized with the Envision-HRP/AP kits (Dako, Ely, UK) with the exception of ESEE 122, which was visualised with the TSA-HRP kit (NEN, Boston, MA, USA).

EDTA, ethylenediamine tetraaceticacid; ON, overnight; RT, room temperature; PC, pressure cooker; MW, microwave; CRUK, Cancer Research, UK.

### Semiquantification

The sections were scored using a semiquantitative system based on the frequency of reactivity of individual parenchymal elements or vessels: no reactivity (−), <1% cells reactive (+), 1–10% cells reactive (++), >10% cells reactive (+++). Two observers performed the scoring over a conference microscope. Evaluation of HIF-1α and HIF-2α was based on the frequency of nuclear and cytoplasmic reactivity,^17^ since previous studies in cancer tissue have shown that the prognostic relevance of a scoring system based on nuclear HIF expression alone was very poor compared with a scoring system that also takes strong cytoplasmic reactivity into account.^18^ DEC-1 score was based on the frequency of nuclear immunoreactivity.^18^ The score for VEGF and its receptors was based on cytoplasmic and plasma membrane reactivity.

Identification of the cellular expression of markers was based on nuclear morphological and dimensional characteristics, as follows. Briefly, neurons were identified in the grey matter by their large nuclei and prominent nucleolus as well as pigment granules, Nissl’s substance or lipofuscin within the cytoplasm. Glia were distinguished from neurons by nuclear phenotype. In general, glial nuclei are smaller, lack nucleoli but often contain clumps of heterochromatin and frequently are found in a satellite position. Blood vessels were identified by their narrow tubular profiles. Layers of the vessel walls could be clearly identified using counterstains with endothelial cells providing the innermost layer and smooth muscle cells, pericytes and perivascular macrophages in the surrounding layers.

### Clinicopathological correlation

The patterns and intensity of immunoreactivity in different cellular groups (as defined above) were correlated with pre-mortem clinical information, including the incidence of systemic and local neurological complications of severe malaria infection before death. These included whether or not the patient suffered from CM before death and the other major systemic complications defining severe malaria according to the WHO criteria,^3^ such as anaemia (haematocrit <20% plus parasitaemia >100 000 μl, haemodynamic shock (systolic blood pressure <80 mmHg), pulmonary oedema, hypoglycaemia (plasma glucose <2.2 mmol/l), jaundice (bilirubin >2.5 mg/dl) or acute renal failure (plasma creatinine >3 mg/dl). Data were analysed using non-parametric tests (Kruskall–Wallis test, Spearman rank correlation and Fisher’s exact test). No adjustments for multiple comparisons were made, although, for the purposes of interpretation and discussion, *P* < 0.01 was regarded as significant.

## Results

### Transcription factors

#### HIF-1α and HIF-2α

**Figure 1:**
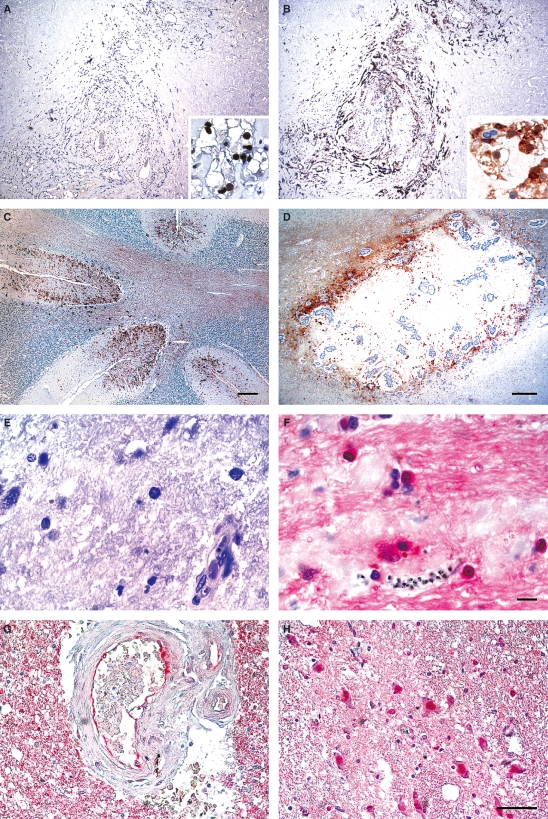
Hypoxia-inducible factor (HIF)-1α, HIF-2α and DEC-1 immunoreactivity of post mortem brain tissue of positive controls (peroxidase, brown) and severe malaria cases (alkaline phosphatase, red). **A**, Nuclear reactivity for HIF-1α in a renal clear cell carcinoma. **B**, Nuclear and cytoplasmic reactivity for HIF-2α in the same renal tumour. **C**, HIF-2α reactivity in a case of generalized hypoxic-ischaemic injury. In the cerebellum there is strong reactivity of HIF-2α on macrophages and glial cells around the depths of the sulci. **D**, HIF-2α reactivity surrounding a metastatic deposit of adenocarcinoma in the brain. Peritumoral macrophages show strong immunopositivity. **E**, HIF-1α is not observed on any of the sections from severe malaria cases. **F**, Nuclear and cytoplasmic HIF-2α is observed at low levels in at least one brain region of all severe malaria cases. **G**, DEC-1 reactivity of endothelial cells in a positive control case with infarction. **H**, Nuclear DEC-1 reactivity in neurons and glial cells in a severe malaria case.

Strong up-regulation of nuclear HIF-1α in peritumoral and intratumoral macrophages was observed in the positive control case of renal clear cell carcinoma (Figure 1A). Up-regulation of nuclear and cytoplasmic HIF-1α was also observed in brain tissue from a fatal case of diffuse ischaemic-hypoxic injury with a prolonged period of perioperative hypoxia. Immunoreactivity for nuclear or cytoplasmic HIF-1α was not observed in any of the malaria cases (Figure 1E).

Strong nuclear and cytoplasmic immunoreactivity was observed for HIF-2α in positive control sections (Figure 1B–D). HIF-2α reactivity was observed in at least one brain region of all severe malaria cases. Reactivity was predominantly cytoplasmic, although occasional HIF-2α-labelled nuclei were observed (Figure 1F). There was a significant increase in the frequency of HIF-2α protein expression associated with the vasculature (including endothelial cells, pericyte/smooth muscle cells and astrocyte endfeet), relative to the non-neurological, multi-organ dysfunction control cases from the UK (referred to as the ‘controls’ for the remainder of the Results section: all brain regions, *P* = 0.005; cortex, *P* = 0.005; diencephalon, *P* = 0.007; brainstem, *P* = 0.01). However, within the severe malaria group there was no difference in the frequency of HIF-2α associated with the vasculature between brain regions or between CM and non-CM cases.

There was no significant increase in the frequency of nuclear and/or cytoplasmic HIF-2α protein expression in neurons or glia, relative to controls. Within the severe malaria group there was more HIF-2α found in glia in the cortex compared with the brainstem (*P* = 0.005), but there was no difference in the frequency of HIF-2α in neurons between brain regions. There was no significant difference in HIF-2α in neurons or glia between CM and non-CM cases.

#### DEC-1 (SHARP2, Stra13)

**Figure 2:**
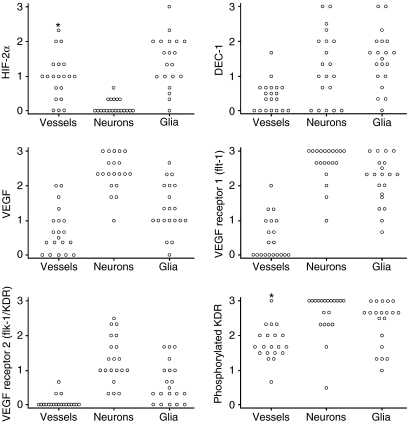
Frequency of immunoreactivity for markers in the brain of fatal cases of severe malaria. The degree of reactivity per tissue section was semiquantified using the following scale: 0, no reactivity; 1, <1% of cells/vessels reactive; 2, 1–10% of cells/vessels reactive; 3, >10% cells/vessels reactive. Each score represents the average of results from three brain regions including the temporal cortex, diencephalon (thalamus, projections of internal capsule) and medulla of the brainstem for an individual patient. *Statistically higher median levels in the severe malaria group compared with non-neurological controls from the UK with multi-organ dysfunction.

Endothelial cells (Figure 1G), glia and neurons (Figure 1H) displayed nuclear accumulation of DEC-1 in sections from severe malaria cases (Figure 2) and the controls. There was a trend for an increased frequency of nuclear DEC-1 protein expression in neurons in severe malaria, relative to controls (*P* = 0.03), but this was not the case for glia or vessels. There was no significant difference in nuclear DEC-1 in neurons, glia or vessels between CM and non-CM cases.

### Vascular endothelial growth factorand its receptors

**Figure 3:**
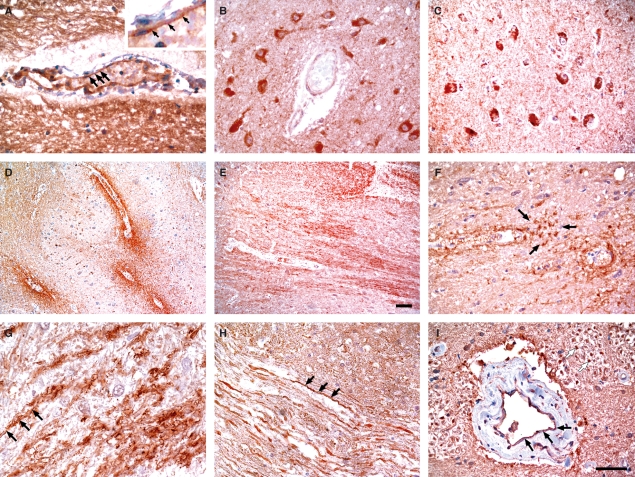
Vascular endothelial growth factor (VEGF), VEGF receptor 1 and activated VEGF receptor 2 (KDR) immunoreactivity in post mortem brain tissue of severe malaria cases and controls. **A**, A vessel with endothelial cells expressing VEGF (arrows). **B**, Endothelial cells, perivascular cells and neurons labelled for VEGF receptor 1. **C**, Neuronal and glial labelling for activated KDR. **D–F**, Different paravascular patterns of immunoreactivity for activated KDR: diffuse (**D**); following white matter tracts (**E**); glial-associated (arrows, **F**). **G–I**, Activated KDR reactivity associated with axonal fibre tracts: longitudinal section showing peri-axonal glial reactivity (arrows) in a severe malaria case (**G**); longitudinal section showing axonal reactivity (arrows) in a case with cerebral infarction (**H**); transverse section showing axonal reactivity (white arrows) in close proximity to a vessel also showing KDR reactivity of endothelial cells (black arrows) and pericyte/smooth muscle cells in a severe malaria case.

**Figure 4:**
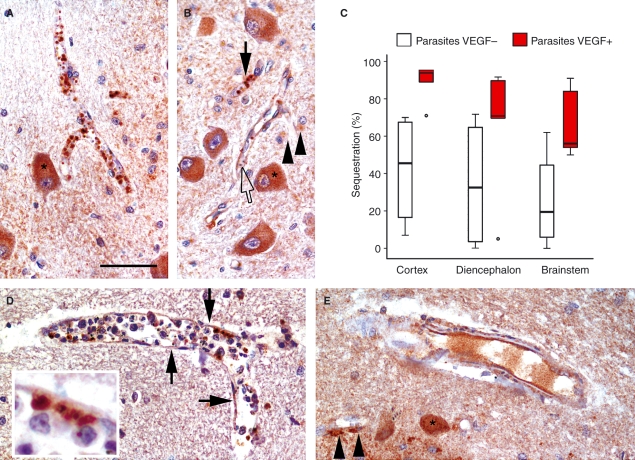
**A**, A vessel containing sequestered malaria parasites that are strongly positive for vascular endothelial growth factor (VEGF). Neurons are also immunolabelled for VEGF in this section (*). **B**, A vessel containing parasites that are VEGF+ (filled arrow). An adjacent vessel with parasites that are not positive for VEGF (empty arrow). This vessel shows light labelling for VEGF on the endothelial cells. Neurons (*) and glia (arrowheads) are also labelled for VEGF in this section. **C**, Graph showing percent sequestration (% vessels with parasite sequestration) in the cortex, diencephalon and brainstem with and without VEGF-labelled parasites. In all but one case, VEGF+ parasites are observed in sections with high sequestration rates. **D**, A large vessel containing numerous intravascular leucocytes. VEGF-labelled parasites appear cytoadhered to the endothelium. Interaction between the endothelium, parasites and monocytes can be observed (see insert, 317% enlargement). Light labelling of the endothelium can be observed in several segments of the vessel (arrows). **E**, A large vessel without intravascular parasites shows strong reactivity for VEGF on endothelial cells and pericytes. The serum is also immunolabelled for VEGF. A smaller vessel in the bottom left corner of the image shows VEGF labelling on perivascular glia (arrowheads). VEGF+ neurons can also be observed (*).

VEGF, VEGFR1 (flt-1), VEGFR2 (flk-1) and phosphorylated VEGFR2 (pKDR) were expressed in both control and severe malaria cases. Expression was associated with vessels, glia and neurons (Figures 2–4).

#### VEGF

Both control brain and severe malaria sections showed a high but heterogeneous frequency of protein expression for VEGF, particularly in neurons (Figures 2 and 4A,B,E). There was no significant increase in the frequency of cytoplasmic VEGF protein expression of neurons, glia or vessels in severe malaria, relative to the controls.

Within the severe malaria group there was a significant difference in cytoplasmic accumulation of VEGF in neurons between brain regions (*P* = 0.005), with a lower frequency found in the cortex compared with the brainstem (*P* = 0.003), and a similar trend was found in the cortex and the diencephalon (*P* = 0.04) but not between diencephalon and brainstem (*P* = 0.15). In contrast, there was no significant difference in the frequency of VEGF protein expression associated with glia or the vasculature between brain regions. There was no significant difference in VEGF in neurons, glia or the vasculature between CM and non-CM cases.

Of interest, we also noted intense immunoreactivity of sequestered *P. falciparum*-infected erythrocytes by VEGF (Figure 4). Of the severe malaria cases, 10 (50%) could be adequately assessed for VEGF reactivity associated with parasite sequestration. Careful examination under oil showed VEGF reactivity in 6/10 cases within the parasites themselves, not the surrounding red cell membrane. This was seen in both CM and non-CM cases. Although other controls showed reactivity of plasma proteins in the vascular space, this pattern appeared specific. Not all cases of malaria showed VEGF immunoreactivity of parasites, and within positive cases not all parasites were reactive. More recently, this finding has been confirmed in cultured parasites, as reported elsewhere.^15^ In all but one case, VEGF+ parasites were observed in sections with high sequestration rates, i.e. >50% vessels sequestered [median (interquartile range) cortex: 94% (89–95%); diencephalon: 71% (70–90%); brainstem: 55% (50–84%)]. Sections containing parasites without VEGF labelling had a lower median percent sequestration rate [cortex: 45.5% (16.5–67.5%); diencephalon: 32.5% (3.5–65%); brainstem: 19.5% (6–44.5%)]. Only cortex showed a statistically significant difference between parasite sequestration rate with VEGF-labelled parasites compared with VEGF^−^ parasites, despite a similar trend in other brain areas (cortex *P* =0.01; diencephalon *P* = 0.16; brainstem *P* = 0.24; Figure 4).

#### VEGF receptor 1 (flt-1)

There was a significant increase in the frequency of flt-1 protein expression in neurons in brainstem (*P* = 0.004), relative to controls. However, there was no significant increase in the frequency of flt-1 protein expression associated with glia or the vasculature relative to controls.

Within the severe malaria group there was a significant difference in flt-1 in neurons with a lower frequency found in the diencephalon compared with the brainstem (*P* = 0.001) but not between cortex and brainstem (*P* = 0.22) or cortex and diencephalon (*P* = 0.13). In contrast, there was no difference in flt-1 in glia or the vasculature between brain regions. There was no significant difference in flt-1 in neurons, glia or the vasculature between CM and non-CM cases.

### VEGF receptor 2 (flK-1) and phosphorylated VEGF receptor 2 (pKDR)

Flk-1 and pKDR were found in parenchymal neurons, glia and associated with the cerebral vasculature in sections from severe malaria cases (Figures 2 and 3C–I) and controls. pKDR was also found on axons (Figure 3H, axons in longitudinal section, black arrows; Figure 3I, axons in cross section, white arrows) and periaxonal glia following axonal tracts (Figure 3E,G).

There was no significant increase in the frequency of flk-1 protein expression associated with neurons, glia or the vasculature relative to controls (Figure 2). Within the severe malaria group there was a significant difference in flk-1 in neurons between brain regions, with a higher frequency found in the brainstem compared with the cortex (*P* = 0.0001) and diencephalon (*P* = 0.007), but not between cortex and diencephalon (*P* = 0.16). In contrast, within the severe malaria group there was no significant difference in flk-1 protein expression associated with glia or the vasculature between brain regions.

Similarly, there was no significant increase in the frequency of pKDR protein expression associated with neurons and glia, relative to controls. However, there was a significant increase in the frequency of pKDR protein expression associated with the vasculature relative to controls when all brain regions were considered (*P* = 0.002) and in the brainstem (*P* = 0.003), but not in the cortex (*P* = 0.56) or diencephalon (*P* = 0.07). Within the severe malaria group there was no significant difference in pKDR protein expression associated with neurons or glia between brain regions. In contrast, there were significant differences in pKDR protein expression associated with the vasculature between brain regions, with a greater frequency of immunoreactivity in the brainstem compared with cortex (*P* = 0.0001) and diencephalon (*P* = 0.0001), but not between the cortex and diencephalon (*P* = 0.10). There was no significant difference in pKDR expression in neurons, glia or the vasculature between CM and non-CM cases.

### Clinicopathological correlations

To investigate the significance of these patterns of marker expression, we performed a detailed clinicopathological correlation between the frequency of marker expression associated with the vasculature, glia and neurons within individual brain regions and the presence of a number of clinical and biochemical parameters using all malaria patients.

Due to the association with the regulation of these markers and hypoxia, the initial analysis focused on complications of malaria infection that may lead to hypoxic damage to the brain and markers of hypoxia. These included percentage of vessels sequestered in the brain, admission peripheral parasite count, hyperparasitaemia, admission/minimum haematocrit/anaemia and plasma lactate, haemodynamic shock, and pulmonary oedema.

Positive correlations were found between the percentage of vessels sequestered and the frequency of HIF-2α labelling associated with the vasculature in the brainstem (*P* = 0.01, *r* = 0.55) and when HIF-2α was expressed as a whole brain average (*P* = 0.002, *r* = 0.64). There was a trend in the diencephalon (*P* = 0.07) but no correlation in the cortex (*P* = 0.33).

Additional parameters analysed included the time to death from admission; plasma levels of glucose; CSF opening pressure, protein and white cell count; and measures of multi-organ disease, in addition to those described above, including jaundice, and acute renal failure. Negative correlations were found between admission plasma glucose levels and frequency of HIF-2α labelling associated with the vasculature in the diencephalon (*P* = 0.009, *r* = −0.61) and when HIF-2α was expressed as a whole brain average (*P* = 0.002, *r* = −0.67). There was a trend in the brainstem (*P* = 0.02) but no correlation in the cortex (*P* = 0.11). There were no correlations between admission plasma glucose and percent parasite sequestration in the brain (*P* > 0.2, for each individual brain area). There were no other statistically significant correlations on univariate analyses.

## Discussion

The aims of this study were to define the endogenous expression of VEGF and its receptors in the brain of cases of fatal severe malaria and identify potential systemic and local regulatory factors. Attempts were made to guard against over-interpretation of our data, by examining changes in malaria cases in comparison with UK control cases who died with multi-organ dysfunction but without structural damage to the brain on standard neuropathological examination.

In severe malaria, the importance of tissue hypoxia is reflected in the consistent observation of elevated concentrations of lactate in plasma on admission, with an increased lactate:pyruvate ratio.^19^ Degradation of HIF-1α under normoxia and its stabilization in hypoxia constitutes a major cellular response during the transition from ambient to low oxygen tension. During normal oxygen supply, HIF-1α is hydroxylated by oxygen-dependent prolyl-4-hydroxylases, ubiquinated and rapidly degraded by the proteosomal system. HIF-1α immunoreactivity of cellular nuclei is thereby often used as a marker of hypoxia. It is also clear that there is heterogeneity in adaptation of altered oxygen tensions between cell types via HIF. For example, an *in vitro* study of how hypoxia affects astrocyte survival demonstrated that HIF-1α protein stabilization occurs only after severe oxygen deprivation of near anoxic (<0.1% O_2_) levels and subsequent reoxygenation (21% O_2_) for 5 h abrogated protein stabilization.^20^ Another study demonstrated minimal HIF-1α protein stabilization in neurons under hypoxic conditions (1% O_2_) with glucose deprivation and a high level of reactive oxygen species (oxidizing environment), which are likely to coincide when there is cerebral ischaemia with disturbed glucose supply and consumption.^21^

In the current study there was strong nuclear immunoreactivity for HIF-1α and HIF-2α in the control tumour cases and brain tissue from a fatal diffuse ischaemic-hypoxic injury. No reactivity for HIF-1α was observed in any of the malaria cases despite multiple attempts using different protocols and antibodies, and despite very good control staining. It therefore appears that widespread persistent stabilization of HIF-1α is not occurring in severe malaria cases. HIF-2α was found at a greater frequency in the vasculature in severe malaria cases compared with non-neurological controls. However, immunolabelling was predominantly found in the cytoplasm, suggesting protein stabilization but a lack of transportation to the site of action. Nevertheless, there was a positive correlation between the percentage of vessels sequestered and a negative correlation with admission plasma glucose levels with the frequency of HIF-2α labelling associated with the vasculature. These findings can be interpreted in several ways. First, a critical threshold of parasite obstruction/hypoxia is not exceeded that would induce HIF expression and/or translocation to the nucleus of cells within the brain. Direct observations of the rectal microcirculation in southeast Asian adults with severe malaria have shown remarkable heterogeneity in microcirculatory blood flow.^22^ Vessels with little or no blood flow were often seen adjacent to vessels with hyperdynamic flow. This would result in a different pattern of pathology from that of large vessel ischaemic damage. Preserved flow in adjacent capillaries may cause a more gradual reduction in blood flow, perhaps with less hypoxia and substrate deprivation. Increased vascular congestion in the cerebral microcirculation with both infected and uninfected erythrocytes is a hallmark of CM, which raises the interesting possibility that there is an inappropriate vasodilation in the brain during CM (Ponsford *et al.*, in preparation). The contribution of neuronal energy requirements to control of local blood flow and vascular architecture in the brain may therefore be disrupted in CM by sequestration, a potential cause of generalized neuronal dysfunction and coma, without producing a picture of hypoxic injury seen in a macrovascular pathology.

The expression of HIFs is strictly regulated, so it cannot be ruled out that peak expression occurred before the patients received treatment or before they died. It should also be considered that destruction of HIFs can occur independently of oxygen requirements via a hydroxylation/ubiquitination/proteosome-independent degradation pathway that is calpain-mediated.^23^ Elevated protein expression and distribution of calpains are observed in these severe malaria cases.^8^ Anticipating that HIF responses may be difficult to detect, we also examined other ‘footprints’ of hypoxia, including downstream markers such as VEGF that can be activated by HIF, and the transcriptional regulator DEC-1 that is regulated by hypoxia and HIF-1α but is more stable. DEC-1 labelling of nuclei was observed in both controls and malaria cases, so HIF-1α stabilization in the nucleus of cells in the brain at some point during the disease course cannot be ruled out. There was a trend for a greater frequency of DEC-1 immunoreactivity in neurons in the brainstem compared with non-neurological controls with multi-organ dysfunction, but no difference was observed between CM and non-CM cases, suggesting a general response to systemic disease.

Studies of plasma levels of VEGF in southeast Asian adults have shown counterintuitive results, with VEGF concentrations lowest in severe malaria and inversely associated with lactate.^13^ The frequency of VEGF protein expression in the brain of the severe malaria cases was heterogeneous within groups, but extensive labelling could be observed in neurons and glial cells in severe malaria cases (Figures 2–4). This was not the case for vascular-associated reactivity with median levels <1% of the total number of vessels. Furthermore, there was no significant difference in the frequency of VEGF labelling of any cell type between controls and severe malaria cases and between CM and non-CM cases. It should be emphasized that our immunohistochemical data indicate the number of cells/vessels containing VEGF protein and is not quantitative for the total amount of VEGF protein produced by the cells. Immunostaining may not detect soluble VEGF diffusing into the parenchyma, and furthermore, no distinctions can be made between systemic sources of VEGF that have gained assess to the brain and been taken up by resident brain cells, or VEGF bound to receptors but produced by another cell locally or at a distance initiating paracrine signalling. Our analysis of the VEGF receptors suffers from similar limitations. However, erythropoietin (EPO) and its receptors also have soluble and cell-associated forms but, in contrast, we have found associations with potential hypoxia-inducing stimuli.^30^ Why these associations were not found with VEGF is uncertain but may reflect the broader range of stimuli that can up-regulate VEGF expression. For example, picolinic acid is a non-hypoxic regulator of VEGF protein expression^24^ that is found in elevated concentrations in the CSF of severe malaria patients.^25^ In addition, there was no immunoreactivity of EPO in parasites, so parasite manipulation of the VEGF response could be inferred. Furthermore, it may be the soluble form of VEGF rather than the cell-associated form that is induced. In a rat model of global ischaemia four VEGF isoforms were induced, but it was the secreted isoform VEGF164 that dominated.^26^

Dealing with such a complex disease and organ as the brain we found the best way to determine whether VEGF signalling is impacting the disease was to use activation state-dependent antibodies. Only cells/vessels with receptors exposed to sufficient levels of ligand to trigger autophosphorylation and subsequent signalling would be visualized. Using a phosphorylated KDR antibody, we have been able to show that the vasculature and cells of the brain parenchyma show a high frequency of VEGF signalling. Similar levels were also found in neurons in the non-neurological controls with multi-organ dysfunction without structural brain damage, suggesting that this is a non-specific response to severe disease. However, we could ascertain that vascular-associated cells and parenchymal cells would be receptive to VEGF signalling through KDR. There were malaria-specific responses with a greater frequency of signalling implied by pKDR immunoreactivity in the vasculature of severe malaria cases compared with UK controls with multi-organ dysfunction, perhaps providing greater cytoprotection in response to a greater level of local microvascular insult. However, there was no correlation with the amount parasite sequestration at death.

Our group has previously shown labelling of parasites for VEGF *in vitro* and *in vivo*.^15^ This study has extended our initial findings by establishing the patterns of VEGF+ parasites in different brain areas within individual patients and by the finding that VEGF+ parasites were primarily observed in cases with high sequestration rates. The latter finding suggests that VEGF is taken up when there is a critical threshold of parasite density within a vessel. This threshold may differ in different brain regions, with the brainstem having the lowest threshold and cortex the highest (Figure 4). Whether VEGF+ parasites are observed sequestered in other organs of these cases is currently under investigation. Since sequestration could be adequately assessed in the brain in only half of the severe malaria cases and since only six of these cases showed VEGF+ parasites, our correlative analysis with clinical and biochemical markers was underpowered. However, it has been established in these cases that cerebral sequestration is inversely proportional to the interval from admission to death and treatment effects (Turner *et al.*, in preparation), so the true incidence of VEGF+ parasite reactivity in the brain during the disease course is probably much higher than this study suggests.

The pathological significance of VEGF+ parasites is unclear. Whether this represents a parasite response to hypoxia is also unknown. The *Plasmodium* parasite is microaerophilic in culture, and there may be some survival advantage to the parasite in maintaining a low oxygen environment during development. No parasite homologues for VEGF were found on a Genbank search of the 3D7 malaria genome. Similarly, the *Plasmodium* parasite genome contains no homologues of HIF-1 or the major proteins controlling oxygen sensing and hypoxic gene regulation in mammalian cells, either at the enzymatic level, including HIF prolyl hydroxylase and factor inhibiting HIF, or the transcriptional level (DEC-1) (observations from GenBank and Plasmodial genome searches). Taken together with our *in vitro* studies, it is most likely that the VEGF is of host origin. At high levels of vessel sequestration there may be increased soluble VEGF production by endothelial cells and perivascular cells as a result of local hypoxia. The sequestered parasites may interrupt autocrine or paracrine signalling in endothelial cells by taking up the VEGF. The uptake of circulating VEGF produced at a distance cannot be ruled out. Whether the parasite utilizes VEGF for its benefit, or modulates signalling through uptake or release of VEGF at schizogony is unknown. However, there is *in vitro* evidence that VEGF can be trophic for parasite growth and partially protect them from the effects of drug treatment.^15^ Impairment of circulating VEGF could contribute to the impaired endothelial nitric oxide production and endothelial dysfunction described in the severe malaria study of Yeo *et al.*^13^

In conclusion, the use of immunohistochemical staining on autopsy tissues from severe malaria cases is not an appropriate way to detect very short-lived and transient patterns of the hypoxia-inducible transcription factor protein expression. A complete lack of HIF induction and/or translocation to the nucleus cannot be ruled out, since we have observed expression of the more stable DEC-1 that is both hypoxia- and HIF-1α-regulated. The frequency of DEC-1 was not malaria specific, with a similar distribution observed in UK controls with multi-organ disease. Although our previous studies in these severe malaria cases have demonstrated hypoxic-associated protein expression of EPO and its receptors,^30^ this was not the case for VEGF and its receptors. This may reflect a broader range of stimuli that can up-regulate VEGF expression. However, EPO was not found to be associated with sequestered malaria parasites. For our interpretation of VEGF signalling we have relied on an activation-specific antibody. Vascular and parenchymal cells would be receptive to the actions of VEGF signalling through KDR. A high frequency of labelling for pKDR could be observed in malaria and controls, suggesting this is a response to severe systemic disease. However, signalling at the vasculature was statistically greater in severe malaria cases, perhaps providing greater cytoprotection in response to a greater level of local injurious insult. The role of the sequestered malaria parasite in these processes requires further investigation.

## Abbreviations:

CM: cerebral malaria
CSF: cerebrospinal fluid
EPO: erythropoietin
HIF: hypoxia-inducible factor
pKDR: phosphorylated VEGFR2
PRBC: parasitized red blood cells
VEGF: vascular endothelial growth factor
VEGFR: VEGF receptor
WHO: World Health Organization
